# A Propensity Score-Matched Cohort Study to Evaluate the Association of Lymph Node Retrieval with Long-Term Overall Survival in Patients with Esophageal Cancer

**DOI:** 10.1245/s10434-020-09142-w

**Published:** 2020-10-16

**Authors:** Leonie R. van der Werf, Elske Marra, Suzanne S. Gisbertz, Bas P. L. Wijnhoven, Mark I. van Berge Henegouwen

**Affiliations:** 1 Department of Surgery, Erasmus University Medical Center, Rotterdam, The Netherlands; 2 Scientific Bureau, Dutch Institute for Clinical Auditing, Leiden, The Netherlands; 3 Department of Surgery, Amsterdam University Medical Centers, University of Amsterdam, Amsterdam, The Netherlands

## Abstract

**Background:**

Previous studies evaluating the association of lymph node (LN) yield and survival presented conflicting results and many may be influenced by confounding and stage migration.

**Objective:**

This study aimed to evaluate whether the quality indicator ‘retrieval of at least 15 LNs’ is associated with better long-term survival and more accurate pathological staging in patients with esophageal cancer treated with neoadjuvant chemoradiotherapy and resection.

**Methods:**

Data of esophageal cancer patients who underwent neoadjuvant chemoradiotherapy and surgery between 2011 and 2016 were retrieved from the Dutch Upper Gastrointestinal Cancer Audit. Patients with < 15 and ≥ 15 LNs were compared after propensity score matching based on patient and tumor characteristics. The primary endpoint was 3-year survival. To evaluate the effect of LN yield on the accuracy of pathological staging, pathological N stage was evaluated and 3-year survival was analyzed in a subgroup of patients with node-negative disease.

**Results:**

In 2260 of 3281 patients (67%) ≥ 15 LNs were retrieved. In total, 992 patients with ≥ 15 LNs were matched to 992 patients with < 15 LNs. The 3-year survival did not differ between the two groups (57% vs. 54%; *p* = 0.28). pN+ was scored in 41% of patients with ≥ 15 LNs versus 35% of patients with < 15 LNs. For node-negative patients, the 3-year survival was significantly better for patients with ≥ 15 LNs (69% vs. 61%, *p* = 0.01).

**Conclusions:**

n this propensity score-matched cohort, 3-year survival was comparable for patients with ≥ 15 LNs, although increasing nodal yield was associated with more accurate staging. In node-negative patients, 3-year survival was higher for patients with ≥ 15 LNs.

**Electronic supplementary material:**

The online version of this article (10.1245/s10434-020-09142-w) contains supplementary material, which is available to authorized users.

Although the extent of lymphadenectomy remains controversial, especially in the era of neoadjuvant therapy, clinical audits often use the number of retrieved lymph nodes (LNs) as a quality indicator for esophageal cancer surgery. In 2013, the percentage of patients with at least 15 retrieved LNs has been introduced as one of the quality indicators in the Dutch Upper Gastrointestinal Cancer Audit (DUCA).^1^ The number of retrieved LNs has increased since the introduction of this quality indicator;^2^ however, it is unclear whether this increase is the result of a more extensive LN dissection or a more extensive pathological examination. Therefore, it might be questioned whether the improvement in LN retrieval since the introduction of this quality indicator in the DUCA has improved locoregional tumor control and thereby might have affected overall survival.

It has been shown that several patient and disease characteristics are associated with the number of retrieved LNs.^2^ Preoperative weight loss of 0–10 kg, low Charlson comorbidity score, and higher clinical N stage were shown to be associated with high LN yield (at least 15 retrieved LNs). When evaluating the association of the number of retrieved LNs with long-term survival, these confounding factors may influence results significantly. Another concern regarding the comparison of outcomes of low versus high LN yield is stage migration.^3^ The accuracy of pathological N stage increases when evaluating more LNs in the pathological examination, and retrieval of more LNs also lowers the risk of leaving positive LNs behind.

The primary aim of this study was to evaluate the association of the quality indicator ‘retrieval of at least 15 LNs’ with long-term survival in a recent national cohort of patients who underwent an esophagectomy after neoadjuvant chemoradiotherapy, with use of a propensity score matching method to minimize the effect of confounding. The secondary aim of this study was to evaluate the association of the quality indicator ‘retrieval of at least 15 LNs’ with the accuracy of pathological staging in this propensity score-matched cohort.

## Methods

### Study Design

For this population-based cohort study, data were retrieved from the DUCA database and a national health care insurance database (Vektis), including date of death.^1^,^4^ All Dutch inhabitants with health care insurance are included in the Vektis database; since health care insurance is obligatory in The Netherlands, almost all Dutch inhabitants (99%) are registered in the Vektis database.^5^ The validity of the merged dataset is estimated at 94%.^6^ For this study, no ethical approval or informed consent was required under Dutch law. The Scientific Bureau and Scientific Committee of the DUCA approved the study design.

### Patient Population

All patients with primary esophageal or esophagogastric junction cancer who underwent neoadjuvant chemoradiotherapy followed by esophagectomy with curative intent in the period between 2011 and 2016 were included. Patients with a resection other than elective were excluded, as were patients with a non-curative esophagectomy (as defined by the surgeon at the end of the operation). Patients were also excluded if data on sex, date of birth, 30-day survival status, number of retrieved LNs, or clinical N category were missing in the DUCA dataset.

### Propensity Score Matching

A propensity score matching method was chosen because correction for confounding factors with a Cox proportional hazard model is not allowed since the assumptions that are needed for this model of proportional hazard over time could not be met in our cohort as the number of LNs increased with time. Propensity score matching was used to create two groups of patients with comparable patient characteristics and disease characteristics. The selection of characteristics that were used for matching was based on the literature. Patients with < 15 LNs were matched to patients with ≥ 15 LNs, on the following characteristics: age, American Society of Anesthesiologists (ASA) score, Charlson comorbidity score, preoperative weight loss, tumor location, clinical T category, clinical N category, clinical M category, histological subtype, and differentiation grade. Characteristics associated with the depending variable (number of retrieved LNs) were not used for matching because of this association; for example, approach (transthoracic vs. transhiatal^7^ and hospital volume^2^). For sensitivity analyses, there were also groups matched for ≥ 10, ≥ 20, and ≥ 30 LNs.

### Outcomes

The primary endpoint of this study was 3-year survival in patients with ≥ 15 and < 15 LNs resected during esophagectomy for esophageal or esophagogastric junction cancer. In the first part of this paper, the 3-year survival was compared between the groups with ≥ 15 and < 15 LNs. For sensitivity analyses, the 3-year survival was also compared for the groups with ≥ 10 versus < 10, ≥ 20 versus < 20, and ≥ 30 versus < 30 LNs. The secondary endpoints in this study were pathological N stage in the groups with ≥ 15 and < 15 LNs. To estimate the accuracy of pathological N staging, in the subgroup of patients with node-negative disease or pN1 disease, the 3-year survival was compared between the groups with ≥ 15 and < 15 LNs. Other N categories were not chosen for evaluation in a subgroup analysis because of heterogenicity within these groups, which might affect outcomes.

### Statistical Analyses

A propensity score-matched analysis was used to balance observed covariates between the group of patients with ≥ 15 retrieved LNs and the group of patients with < 15 retrieved LNs. The groups were matched using the nearest-neighbor method with a caliper of 0.20. Balances in patient and disease characteristics between the groups were measured using the standardized mean difference; differences of more than 10% represent inadequate balance. Overall survival of the groups was analyzed using Kaplan–Meier survival curves with 95% confidence intervals (CIs) and 3-year survival rate. These outcomes were compared using log-rank analyses. The pathological N stages were compared between the two groups using *χ*^2^ analyses. Missing items were categorized in a separate group. For sensitivity analyses, comparisons were also made for groups with ≥ 10 versus < 10, ≥ 20 versus < 20, and ≥ 30 versus < 30 LNs. For all analyses, statistical significance was defined as *p* < 0.05. All analyses were performed using SPSS version 24 (IBM Corporation, Armonk, NY, USA) and R studio version 1.1.456 (RStudio, Inc, packages: ‘MatchIt’ and ‘optmatch’).

## Results

### Study Population

**Fig. 1:**
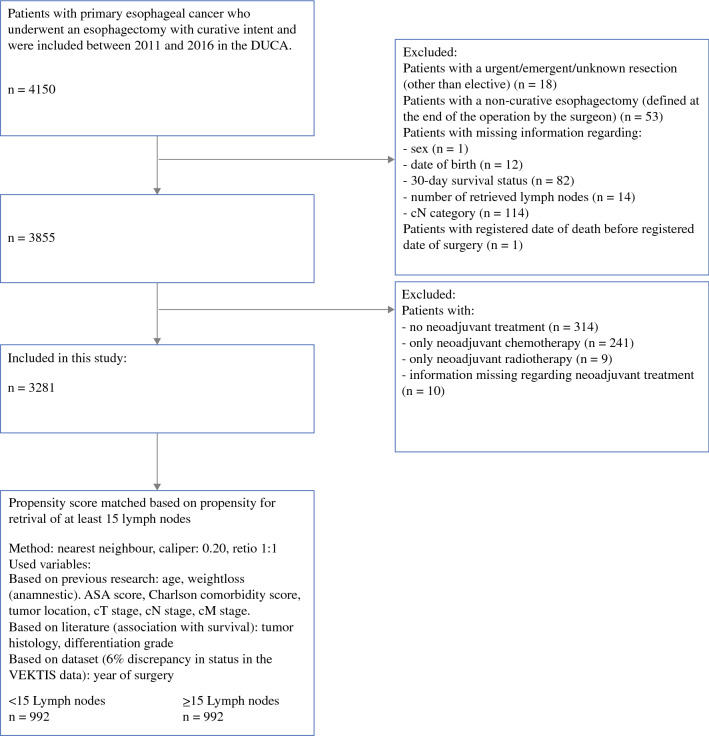
Inclusion and exclusion criteria and propensity score matching. *DUCA* Dutch Upper Gastrointestinal Cancer Audit, *ASA* American Society of Anesthesiologists

A total of 3281 esophageal cancer patients underwent neoadjuvant chemoradiotherapy followed by curative esophagectomy between 2011 and 2016 and were eligible for this study according to the inclusion and exclusion criteria (Fig. 1). Retrieval of at least 15 LNs was achieved in 2260 (67%) patients.

With propensity score matching, 992 patients with < 15 retrieved LNs were matched to 992 patients with ≥ 15 retrieved LNs. Patient, disease, and treatment characteristics are shown in Table 1.

**Table 1:** Basic characteristics of the total cohort and the propensity score-matched cohort

Total cohort	Propensity score-matched cohort					
	<15 lymph nodes	≥15 lymph nodes	SMD	<15 lymph nodes	≥15 lymph nodes	SMD
Total	1021	2260		992	992	
Sex						
Male	809 (79.2)	1756 (77.7)	0.037	783 (78.9)	776 (78.2)	0.017
Female	212 (20.8)	504 (22.3)		209 (21.1)	216 (21.8)	
Age, years [mean (SD)]	65.50 (8.62)	64.25 (8.77)	0.144	65.28 (8.57)	65.30 (8.63)	0.002
ASA score						
I	156 (15.3)	422 (18.7)	0.124	155 (15.6)	154 (15.5)	0.052
II	622 (60.9)	1372 (60.7)		607 (61.2)	601 (60.6)	
III	231 (22.6)	453 (20.0)		220 (22.2)	230 (23.2)	
IV	3 (0.3)	6 (0.3)		3 (0.3)	1 (0.1)	
Unknown	9 (0.9)	7 (0.3)		7 (0.7)	6 (0.6)	
Charlson comorbidity score						
0	483 (47.3)	1153 (51.0)	0.091	473 (47.7)	471 (47.5)	0.064
1	258 (25.3)	572 (25.3)		251 (25.3)	275 (27.7)	
2+	280 (27.4)	535 (23.7)		268 (27.0)	246 (24.8)	
Body mass index						
< 20	69 (6.8)	145 (6.4)	0.07	68 (6.9)	60 (6.0)	0.056
20–24	380 (37.2)	862 (38.1)		366 (36.9)	374 (37.7)	
25–29	387 (37.9)	890 (39.4)		377 (38.0)	392 (39.5)	
29+	170 (16.7)	342 (15.1)		166 (16.7)	153 (15.4)	
Unknown	15 (1.5)	21 (0.9)		15 (1.5)	13 (1.3)	
Weight loss (anamnestic)						
< 10 kg	731 (71.6)	1763 (78.0)	0.195	714 (72.0)	723 (72.9)	0.045
10.1–15 kg	77 (7.5)	139 (6.2)		74 (7.5)	65 (6.6)	
> 15 kg	40 (3.9)	113 (5.0)		39 (3.9)	44 (4.4)	
Unknown	173 (16.9)	245 (10.8)		165 (16.6)	160 (16.1)	
Tumor location						
Proximal/mid thoracic	105 (10.3)	357 (15.8)	0.209	105 (10.6)	111 (11.2)	0.053
Distal	663 (64.9)	1490 (65.9)		654 (65.9)	629 (63.4)	
Gastroesophageal junction	253 (24.8)	413 (18.3)		233 (23.5)	252 (25.4)	
cT category						
cT0-1	17 (1.7)	26 (1.2)	0.073	16 (1.6)	10 (1.0)	0.059
cT2	212 (20.8)	425 (18.8)		202 (20.4)	197 (19.9)	
cT3	736 (72.1)	1679 (74.3)		720 (72.6)	727 (73.3)	
cT4	35 (3.4)	74 (3.3)		33 (3.3)	37 (3.7)	
cTx/Unknown	21 (2.1)	56 (2.5)		21 (2.1)	21 (2.1)	
cN category						
cN0	365 (35.7)	724 (32.0)	0.104	350 (35.3)	352 (35.5)	0.048
cN1	446 (43.7)	987 (43.7)		435 (43.9)	448 (45.2)	
cN2	177 (17.3)	464 (20.5)		174 (17.5)	164 (16.5)	
cN3	22 (2.2)	61 (2.7)		22 (2.2)	17 (1.7)	
cN4	11 (1.1)	24 (1.1)		11 (1.1)	11 (1.1)	
cM category						
cM0	990 (97.0)	2208 (97.7)	0.051	961 (96.9)	968 (97.6)	0.048
cM+	6 (0.6)	13 (0.6)		6 (0.6)	6 (0.6)	
cMx/Unknown	25 (2.4)	39 (1.7)		25 (2.5)	18 (1.8)	
Histological type						
Adenocarcinoma	728 (71.3)	1554 (68.8)	0.073	705 (71.1)	721 (72.7)	0.075
Squamous cell carcinoma	164 (16.1)	423 (18.7)		162 (16.3)	149 (15.0)	
Other	19 (1.9)	43 (1.9)		18 (1.8)	15 (1.5)	
Not applicable	81 (7.9)	183 (8.1)		80 (8.1)	88 (8.9)	
Unknown	29 (2.8)	57 (2.5)		27 (2.7)	19 (1.9)	
Differentiation grade						
Well/moderate	358 (35.1)	868 (38.4)	0.092	348 (35.1)	350 (35.3)	0.059
Poor	276 (27.0)	600 (26.5)		269 (27.1)	277 (27.9)	
Not judgeable	184 (18.0)	340 (15.0)		176 (17.7)	155 (15.6)	
Unknown	203 (19.9)	452 (20.0)		199 (20.1)	210 (21.2)	
Year of surgery						
2011	202 (19.8)	189 (8.4)	0.482	179 (18.0)	146 (14.7)	0.145
2012	187 (18.3)	287 (12.7)		181 (18.2)	193 (19.5)	
2013	188 (18.4)	322 (14.2)		188 (19.0)	169 (17.0)	
2014	150 (14.7)	429 (19.0)		150 (15.1)	190 (19.2)	
2015	167 (16.4)	504 (22.3)		167 (16.8)	155 (15.6)	
2016	127 (12.4)	529 (23.4)		127 (12.8)	139 (14.0)	
Resection margins (pathological)						
pR0	962 (94.2)	2152 (95.2)	0.047	935 (94.3)	944 (95.2)	0.05
pR+	47 (4.6)	89 (3.9)		46 (4.6)	41 (4.1)	
Unknown	12 (1.2)	19 (0.8)		11 (1.1)	7 (0.7)	
*Other characteristics (matching not based on these variables)*						
Procedure						
TTE (thoracic open)	149 (14.6)	402 (17.8)	0.843	148 (14.9)	167 (16.8)	0.7
TTE (thoracic MI)	308 (30.2)	1426 (63.1)		307 (30.9)	592 (59.7)	
THE (open)	441 (43.2)	296 (13.1)		417 (42.0)	163 (16.4)	
THE (MI)	116 (11.4)	133 (5.9)		113 (11.4)	68 (6.9)	
Unknown	7 (0.7)	3 (0.1)		7 (0.7)	2 (0.2)	
Annual hospital volume						
0–25	226 (22.1)	187 (8.3)	0.446	223 (22.5)	86 (8.7)	0.426
26–50	527 (51.6)	1304 (57.7)		511 (51.5)	578 (58.3)	
50+	250 (24.5)	765 (33.8)		240 (24.2)	324 (32.7)	
Stopped with esophageal surgery before 2014	18 (1.8)	4 (0.2)		18 (1.8)	4 (0.4)	

Data are expressed as *n* (%) unless otherwise specified

*SMD* standard mean difference, *SD* standard deviation, *ASA* American Society of Anesthesiologists, *TTE* trans-thoracic esophagectomy, *THE* transhiatal esophagectomy, *MI* myocardial infarction

### Overall Survival Outcomes between Patients with ≥ 15 versus < 15 Retrieved Lymph Nodes

**Fig. 2:**
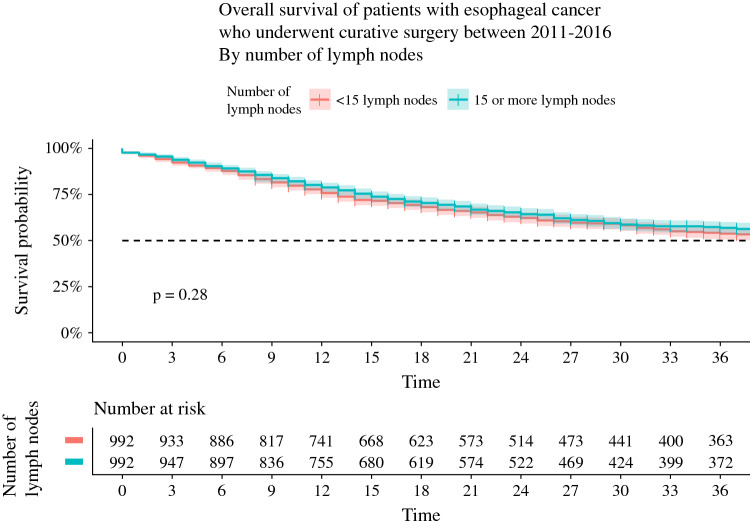
Overall survival curves with 95% confidence interval of the subgroups with ≥ 15 LNs versus < 15 LNs in the propensity matched cohort. *LNs* lymph nodes

The overall survival curves in the propensity score-matched cohort are presented in Fig. 2. The 3-year survival was not significantly different between the group of patients with ≥ 15 retrieved LNs and patients with < 15 retrieved LNs (57% vs. 54%; *p* = 0.28). In sensitivity analyses, there were also no differences in 3-year survival when comparing patients with ≥ 10 LNs versus patients with < 10 LNs (52% vs. 54%; *p* = 0.31), patients with ≥ 20 LNs versus patients with < 20 LNs (55% vs. 55%; *p* = 0.88), and patients with ≥ 30 LNs versus patients with < 30 LNs (59% vs. 59%; *p* = 0.54) [electronic supplementary Figs. 1, 2, and 3].

### Pathological Staging

In the propensity score-matched cohort, the clinical T and N stages were well-balanced between the groups with ≥ 15 and < 15 retrieved LNs. After pathological staging, patients with ≥ 15 retrieved LNs were staged with higher N stages (*p* < 0.001) [Table 2].

**Table 2:** Pathological staging of the subgroups with ≥ 15 versus < 15 LNs in the propensity matched cohort

Total	<15 lymph nodes	≥15 lymph nodes	*p* Value
	992	992	
pT stage			
pT0-1	393 (39.6)	374 (37.7)	0.393
pT2	223 (22.5)	206 (20.8)	
pT3	332 (33.5)	366 (36.9)	
pT4	2 (0.2)	5 (0.5)	
pTx	42 (4.2)	41 (4.1)	
pN stage			
pN0	647 (65.2)	583 (58.8)	<0.001
pN1	202 (20.4)	208 (21.0)	
pN2	90 (9.1)	108 (10.9)	
pN3	15 (1.5)	57 (5.7)	
pNx	38 (3.8)	36 (3.6)	

Data are expressed as *n* (%)

**Fig. 3:**
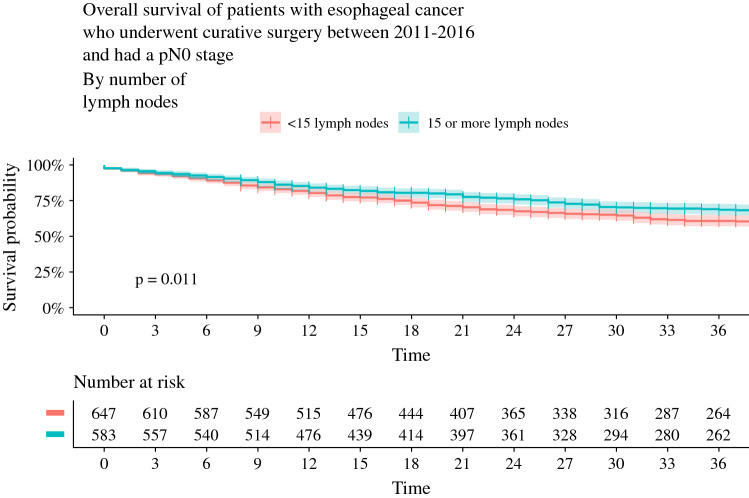
Overall survival curves with 95% confidence interval of the subgroups with < 15 LNs versus ≥ 15 LNs in the propensity matched cohort with pN0 stage disease. *LNs* lymph nodes

**Fig. 4:**
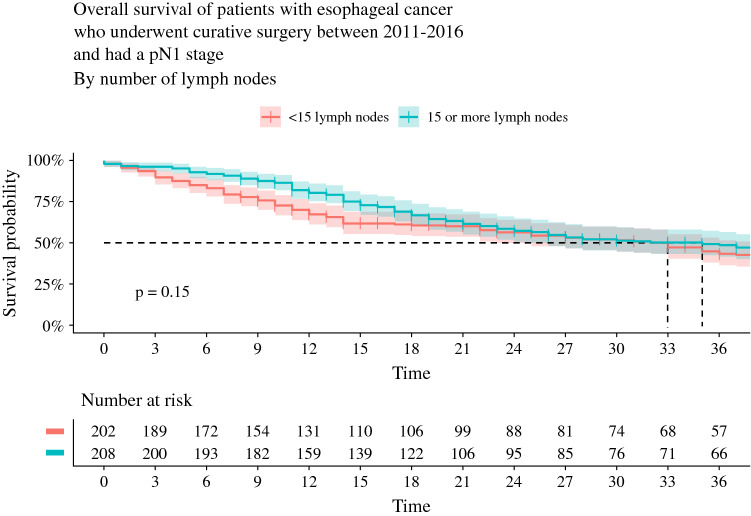
Overall survival curves with 95% confidence interval of the subgroups with ≥ 15 LNs versus < 15 LNs in the propensity matched cohort with pN1 stage disease. *LNs* lymph nodes

The 3-year survival in the subgroup of patients with pathological N0 status was significantly higher for patients with ≥ 15 retrieved LNs compared with patients with < 15 LNs (69% vs. 61%, *p* = 0.01) [Fig. 3]. For the subgroup of patients with pathological N1 status, 3-year survival was not significantly different between the groups with ≥ 15 and < 15 LNs (49% vs. 43%; *p* = 0.15) [Fig. 4].

## Discussion

This study investigated whether the quality indicator ‘retrieval of at least 15 LNs’ was associated with better long-term survival and more accurate pathological staging in patients with esophageal cancer treated with neoadjuvant chemoradiotherapy and resection. The results of this study showed that there was no difference in 3-year survival between patients with retrieval of at least 15 LNs versus patients with retrieval of < 15 LNs. In addition, retrieval of at least 10, 20, or 30 LNs was not associated with better 3-year survival compared with patients with fewer LNs (< 10, < 20, and < 30, respectively); however, retrieval of at least 15 LNs was associated with more accurate pathological staging. Positive LNs were found more often in patients with at least 15 retrieved LNs, leading to higher pathological N stages. Furthermore, the 3-year survival in the subgroup of patients with pathological node-negative disease was significantly higher for patients with at least 15 LNs compared with patients with < 15 LNs. These findings support the idea of stage migration; patients with low LN retrieval are likely understaged because positive LNs have been left behind in the resection specimen or may have been left behind in the patient. This may explain the lower 3-year survival for patients with pathological node-negative disease with < 15 LNs compared with patients with at least 15 retrieved LNs.

The therapeutic value of a higher number of retrieved LNs after neoadjuvant therapy is a controversial issue in cancer surgery. For esophageal cancer, many papers have been published on this topic and most studies show an association between the number of nodes retrieved and survival.^8^,^9^ The findings of the current study show this relationship only for patients with pathological node-negative disease; in the total group, no relation between LN retrieval and survival was seen. A possible explanation may be patient selection; the patient cohort that is selected covers a more recent period than most other studies. Dutch institutions have started various improvement processes in recent years. The number of LNs resected was implemented as a quality indicator in 2013, which has resulted in an increase in the number of retrieved LNs reported^10^ The increase in reported LN yield may not only be an effect of more extended LN dissections but may also be due to more detailed pathological examination; therefore, the extra number of examined and counted LNs does not automatically imply an extended lymphadenectomy.

The outcome that there was no survival difference between the two groups may also be due to recent improvements in preoperative and intraoperative imaging, which may lead to better-targeted lymphadenectomy. Better-targeted lymphadenectomy might ensure the quality of LN dissection, but is not necessarily reflected in the high number of LNs. In The Netherlands, the use of preoperative positron emission tomography/computed tomography (PET/CT) and endoscopic ultrasound (EUS) for clinical staging increased. In 2017, a PET/CT was performed in 93% of patients, an EUS was performed in 67% of patients, and an EUS with biopsy was performed in 18% of patients.^11^ For patients with esophageal squamous cell carcinoma, a recent meta-analysis showed that the pooled sensitivity of PET/CT for detection of regional LN metastasis was 66% (95% CI 66–78%).^12^ Another recent meta-analysis evaluated the sensitivity of PET/CT and EUS for detecting residual disease after neoadjuvant chemoradiotherapy at the primary tumor site or regional LNs.^13^ For PET/CT, the sensitivity rates for detection of residual disease in LNs ranged between 0 and 65%. Due to the low number of studies evaluating the sensitivity of PET/CT for detection of residual disease in LNs, the authors could not determine the pooled sensitivity for PET/CT. For EUS, the pooled sensitivity for detection of residual disease in LNs was 68% (95% CI 54–80%).

In future, intraoperative imaging as fluorescence imaging may also help to identify affected LNs.^14^

A limitation of this study was that with propensity score matching, it was possible to only compare two groups and it was not possible to use the number of LNs as a continuous outcome. Therefore, identifying an optimal number of LNs was not possible. Additionally, not analyzing the number of LNs as a continuous outcome could have been the reason that no survival difference was seen between patients with a low versus high number of retrieved LNs. Another limitation is that this study did not include which LN stations were dissected, therefore it is not known whether these LN stations influenced survival.

A further study with more focus on the extent of LN dissection is needed. It would be desirable to identify an optimal number of LNs that should be removed, or to identify which LN stations should be dissected. For this purpose, the TIGER study is under way;^15^ the aim of this international observational cohort study is to evaluate the distribution of LN metastases in esophageal carcinoma. In 50 centers, specimens of patients following transthoracic esophagectomy with a two- or three-field lymphadenectomy will be evaluated by a pathologist. The distribution of LN metastases will be evaluated in relation to tumor histology, tumor location, invasion depth, number of LNs and LN metastases, preoperative diagnostics, neoadjuvant therapy, and (disease-free) survival.

Taken together, although the findings of the current study did not show that retrieval of at least 15 LNs was associated with improved 3-year survival, it did show that it was associated with more accurate pathological staging. Since accurate staging is important to determine prognosis, and therefore contributes to better quality of care, it can be concluded that ‘retrieval of at least 15 LNs’ is a relevant quality indicator.

## Electronic supplementary material

Below is the link to the electronic supplementary material.

Supplementary material 1 (PDF 521 kb)
