# Supplementary material for: A Propensity Score-Matched Cohort Study to Evaluate the Association of Lymph Node Retrieval with Long-Term Overall Survival in Patients with Esophageal Cancer
Source: Ann Surg Oncol. 2020 Oct 16;28(1):133–41. doi: 10.1245/s10434-020-09142-w (PMC7752882; doi:10.1245/s10434-020-09142-w)
Supplement: Supplementary file 1 — Supplementary material 1 (PDF 521 kb) [file 10434_2020_9142_MOESM1_ESM.pdf]

Supplementary figures

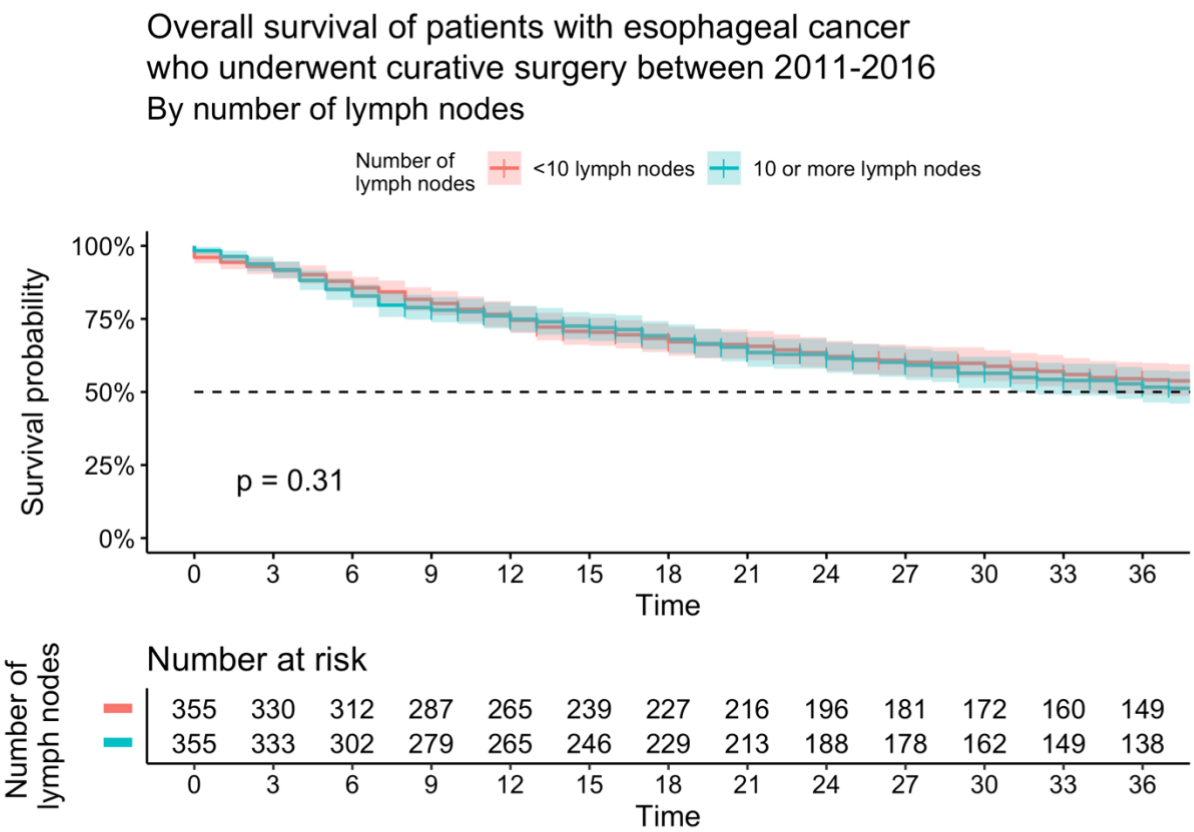

Supplementary figure 1: Overall survival curves with 95% confidence interval of the subgroups ≥10 LNs versus <10 LNs in the propensity matched cohort.

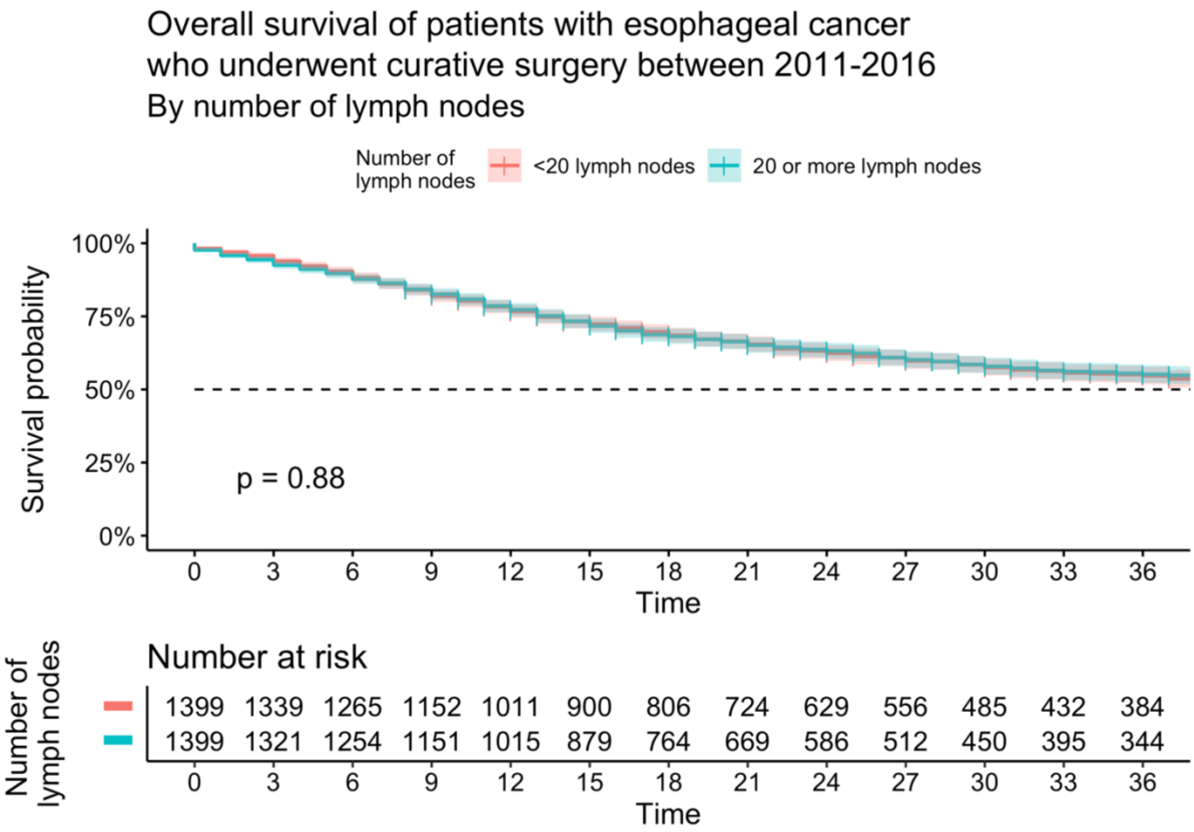

Supplementary figure 2: Overall survival curves with 95% confidence interval of the subgroups ≥20 LNs versus <20 LNs in the propensity matched cohort.

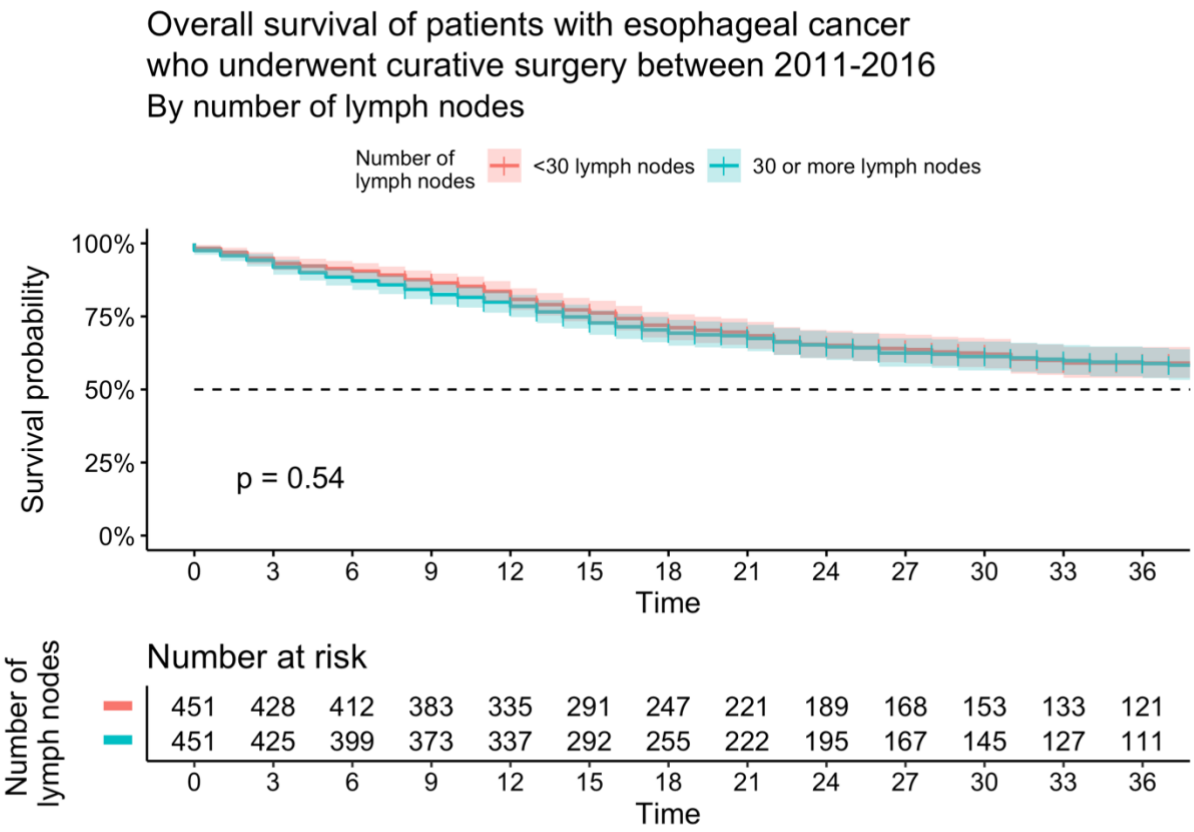

Supplementary figure 3: Overall survival curves with 95% confidence interval of the subgroups ≥30 LNs versus <30 LNs in the propensity matched cohort.
